# A Phase 1/2 trial of SRA737 (a Chk1 inhibitor) administered orally in patients with advanced cancer

**DOI:** 10.1038/s41416-023-02279-x

**Published:** 2023-04-29

**Authors:** Rebecca Kristeleit, Ruth Plummer, Robert Jones, Louise Carter, Sarah Blagden, Debashis Sarker, Tobias Arkenau, Thomas R. Jeffry Evans, Sarah Danson, Stefan N. Symeonides, Gareth J. Veal, Barbara J. Klencke, Mark M. Kowalski, Udai Banerji

**Affiliations:** 1 Guy’s and St Thomas’ NHS Foundation Trust, London, UK; 2 Newcastle University and Newcastle Hospitals NHS Trust, Newcastle Upon Tyne, UK; 3 Velindre School of Medicine, Cardiff University, and Velindre University NHS Trust, Cardiff, UK; 4 Division of Cancer Sciences, The University of Manchester and The Christie NHS Foundation Trust, Manchester, UK; 5 Early Phase Clinical Trials Unit, Churchill Hospital, Oxford University Hospital NHS Trust, Oxford, UK; 6 King’s College London and Guy’s Hospital, London, UK; 7 Sarah Cannon Research Institute, London, UK; 8 The Beatson West of Scotland Cancer Centre and the University of Glasgow, Glasgow, UK; 9 Sheffield ECMC, Department of Oncology and Metabolism, University of Sheffield, and Sheffield Teaching Hospital NHS Foundation Trust, Sheffield, UK; 10 Edinburgh ECMC, Institute of Genetics & Cancer, University of Edinburgh, Edinburgh Cancer Centre, Edinburgh, UK; 11 Newcastle University Centre for Cancer, Translational and Clinical Research Institute, Newcastle upon Tyne, UK; 12 Sierra Oncology Inc., San Mateo, CA USA; 13 The Institute of Cancer Research and The Royal Marsden Hospital NHS Foundation Trust, London, UK

**Keywords:** Drug development, Drug development, Cancer genomics

## Abstract

**Background:**

This was a first-in-human Phase 1/2 open-label dose-escalation study of the novel checkpoint kinase 1 (Chk1) inhibitor SRA737.

**Methods:**

Patients with advanced solid tumours enrolled in dose-escalation cohorts and received SRA737 monotherapy orally on a continuous daily (QD) dosing schedule in 28-day cycles. Expansion cohorts included up to 20 patients with prospectively selected, pre-specified response predictive biomarkers.

**Results:**

In total, 107 patients were treated at dose levels from 20–1300 mg. The maximum tolerated dose (MTD) of SRA737 was 1000 mg QD, the recommended Phase 2 dose (RP2D) was 800 mg QD. Common toxicities of diarrhoea, nausea and vomiting were generally mild to moderate. Dose-limiting toxicity at daily doses of 1000 and 1300 mg QD SRA737 included gastrointestinal events, neutropenia and thrombocytopenia. Pharmacokinetic analysis at the 800 mg QD dose showed a mean *C*_min_ of 312 ng/mL (546 nM), exceeding levels required to cause growth delay in xenograft models. No partial or complete responses were seen.

**Conclusions:**

SRA737 was well tolerated at doses that achieved preclinically relevant drug concentrations but single agent activity did not warrant further development as monotherapy. Given its mechanism of action resulting in abrogating DNA damage repair, further clinical development of SRA737 should be as combination therapy.

**Clinical trial registration:**

Clinicaltrials.gov NCT02797964.

## Background

Checkpoint kinase 1 (Chk1) is a serine/threonine kinase encoded by the CHEK1 gene [1]. It is a crucial component of the ATR-Chk1-Wee1 axis of DNA damage response [2] and homozygous knockout of Chk1 is embryonically lethal [3]. It plays a key role in regulation of the cell cycle, in particular at the G2/M checkpoint, where it prevents cancer cells with extrinsic DNA damage caused by factors such as chemotherapy or radiotherapy from undergoing mitosis, thereby allowing time for DNA repair. Importantly, a further function of Chk1 is to prevent cancer cells with intrinsic DNA damage due to rapid growth such as mutated oncogenes, defective G1/S checkpoints due to TP53 mutations, or cyclin E amplifications from entering mitosis, allowing for DNA repair [4]. SRA737 (Sierra Oncology, Inc., San Mateo, California, USA) is a novel orally bioavailable selective Chk1 inhibitor that has shown single-agent preclinical activity in MYC-amplified models of neuroblastoma [5] and lymphoma [6] in addition to cancer cells with loss of B-family DNA polymerase function [7]. SRA737 is hypothesized to have antitumour activity when administered as a single agent.

## Methods

### Study design

The objectives of this first-in-human, Phase 1/2, open-label, dose-escalation study of SRA737 as a single agent were to establish the safety profile, define the maximum tolerated dose (MTD) and the recommended Phase 2 dose (RP2D) and schedule, and to evaluate preliminary efficacy in prospectively selected genetically defined patients enrolled in indication-specific expansion cohorts. Secondary objectives included defining the pharmacokinetic (PK) profile of SRA737.

The study used an accelerated titration design in dose escalation beginning with single subject dose-level cohorts. Doubling of the SRA737 dose was permitted in the subsequent cohort if a dose level was determined to be safe by the cohort review committee consisting of the lead investigator, study investigators representing the site(s) currently enrolling patients, and representatives of the study sponsor. In the event of any SRA737-related adverse event Grade 2 or greater toxicity during Cycle 1, the cohort was to be expanded to 3 to 6 subjects. Thereafter the study followed a rolling 6 design wherein once the first subject completed the 7-day observation period following the first dose of SRA737, subsequent subjects in that cohort started treatment. In the absence of a dose-limiting toxicity (DLT), the dose of SRA737 was escalated in less than 100% (typically 25–75%) increments. Enrolment of expansion cohorts at the highest dose level determined to be safe and tolerable was initiated prior to the completion of dose escalation and determination of MTD or RP2D.

The trial (ClinicalTrials.gov identifier NCT02797964) was carried out in 15 centres in the United Kingdom between July 18, 2016 and October 28, 2019.

Research ethics committees approved the study protocol before initiation of patient enrolment, and all patients provided written informed consent prior to study enrolment in accordance with the Declaration of Helsinki, the International Conference on Harmonisation Guidelines for Good Clinical Practice, and applicable local regulations.

### Participants

Participants in the dose-escalation phase included patients with solid tumours who had relapsed or were progressing after having had standard-of-care chemotherapy. They were required to have World Health Organization (WHO) performance status 0–1 and organ function within limits of standard Phase 1 studies (Supplementary Methods).

Tumour type-specific expansion cohorts recruited prospectively identified genetically-defined patients who met the above eligibility criteria and had one of the following 6 tumour types: colorectal cancer (CRC); high-grade serous ovarian cancer (HGSOC) further designated as those with or without CCNE1 gene amplification and those with CCNE1 gene amplification (or alternative genetic alteration with similar functional effect); metastatic castration-resistant prostate cancer (mCRPC); non-small cell lung cancer (NSCLC); or head and neck squamous cell carcinoma (HNSCC) or squamous cell cancer of the anus (SCCA). Patients in these cohorts had their tumours (archival or fresh tissue) prospectively sequenced and were required to have aberrations in one or more of the following categories: (a) tumour suppressor genes regulating G1 such as RB1, TP53, and for patients with HNSCC or SCCA, positive human papillomavirus (HPV) status was also acceptable for eligibility; (b) DNA damage response pathway ATM, BRCA1, BRCA2; (c) genetic indicators of replicative stress such as gain of function/amplification of CHK1 or ATR; d) oncogenic driver such as KRAS or MYC (Supplementary Table 1).

### Treatment

SRA737 is a novel, weakly basic compound presented for oral clinical administration as its citrate salt. The molecular weight of SRA737 is 379.34 Da for the free base and 571.46 Da for the citrate salt. SRA737 capsules were be taken on an empty stomach, at the same time each day. Subjects were instructed to fast for 2 h before administration and for 1 h after administration. The instructions were to be followed throughout the study drug administration period including the PK assessment on Day −7 to −4. It was also instructed that the administration of antacids or H2 antagonists should occur 4 h before or 2 h after administration of SRA737. This latter instruction was due to the fact that preliminary in vitro studies suggest that the SRA737 citrate drug product may possess some minimal to moderate pH-dependent solubility over the physiological range.

A single dose of SRA737 was given at one visit on Day −7 to Day −4 (prior to the start of Cycle 1) for PK assessments. SRA737 was then administered orally on a continuous daily dosing schedule in 28-day cycles. An accelerated titration design was used with single-patient dose-escalation cohorts starting with 20 mg SRA737 administered once daily (QD) in the first cohort, with dose doubling in subsequent single-patient cohorts. If a patient had SRA737-related Grade 2 toxicity in a dose level cohort during Cycle 1, that cohort was expanded to 3 to 6 patients, and subsequent cohorts followed a rolling six design.

Patients were assessed for DLT from the first SRA737 dose (Day −7 to Day −4) until the end of Cycle 1 (up to 35 days). A DLT was defined as any highly probable or probably treatment-related event that met protocol-specified DLT criteria, including any drug-related toxicity that led to an inability to receive at least 75% of the planned dose in the first cycle (Supplementary Methods).

Expansion cohorts including up to 20 patients with one of the 6 specified indication-specific tumour types, were treated at SRA737 doses selected by the cohort review committee based on all available safety and PK data; expansion doses were at or below the maximum tolerated dose (MTD) from the dose-escalation phase. Patients could continue treatment until disease progression or discontinuation from study due to unacceptable toxicity, investigator/sponsor decision, or withdrawal of consent.

### Assessments

Safety assessments including adverse events, laboratory parameters, electrocardiograms (ECGs), and echocardiograms were conducted throughout treatment and until 30 days after the last study treatment or initiation of new anticancer treatment. Toxicity was recorded using National Cancer Institute Common Terminology Criteria for Adverse Events (NCI CTCAE) version 4.03.

Serial sampling of blood for PK assessment was conducted before and after dosing with single-agent SRA737 (9 time points over 48 h) on Day −7 to Day −4 and on Cycle 1 Day 22; in addition, samples were drawn pre-dose on Days 1, 8, and 15 of Cycle 1, and pre-dose on Day 1 of each subsequent cycle. Plasma SRA737 concentrations were quantified using a fully validated liquid chromatography-mass spectrometry assay with a lower limit of quantitation of 5 ng/ml as previously described [8].

Cardiac assessments included centrally-assessed serial ECGs in Cycle 1 used for evaluation of QTc. In addition, locally-read ECGs for safety assessment were also available for central assessment where clinically indicated. Troponin T or I assessments and echocardiogram were locally assessed.

Genetic tumour profiling for eligibility was performed either from tumour tissue (FoundationOne^®^ CDx Panel, Foundation Medicine, Inc., Cambridge, MA) or circulating tumour DNA (ctDNA). The FoundationOne^®^ panel is a qualitative next-generation sequencing-based in vitro diagnostic test that uses targeted high throughput hybridization-based capture technology for detection of substitutions, insertion and deletion alterations (indels), and copy number alterations in 324 genes and select gene rearrangements, as well as genomic signatures including microsatellite instability (MSI) and tumour mutational burden (TMB) using DNA isolated from formalin-fixed paraffin-embedded (FFPE) tumour tissue specimens, including archival specimens.

Radiologic tumour assessments were carried out every 8 weeks, and clinical evaluations and serum tumour markers assessed every 4 weeks. Tumours were assessed using the Response Evaluation Criteria in Solid Tumors version 1.1 (RECIST 1.1). Clinical response data were summarized in cohorts defined by tumour type (CRC, HGSOC, NSCLC, mCRPC, HNSCC, or other disease).

### Statistical analysis

The Safety Evaluable population included all patients who received at least one dose of SRA737. The Response Evaluable population included patients who had measurable disease at baseline, received at least 75% of planned SRA737 doses in Cycle 1 and had at least one post-baseline disease assessment or discontinued treatment due to disease progression, adverse event, or death.

Safety and response variables were summarized using descriptive statistics and PK parameters were determined using non-compartmental methods. A concentration-effect model was used to evaluate the relationship between plasma SRA737 concentrations and the mean change from baseline in QT interval corrected for heart rate using Fridericia’s formula (QTcF) in time-matched ECG measurements and PK samples collected on Day −7 to Day −4 and during Cycle 1.

## Results

### Demography

**Fig. 1:**
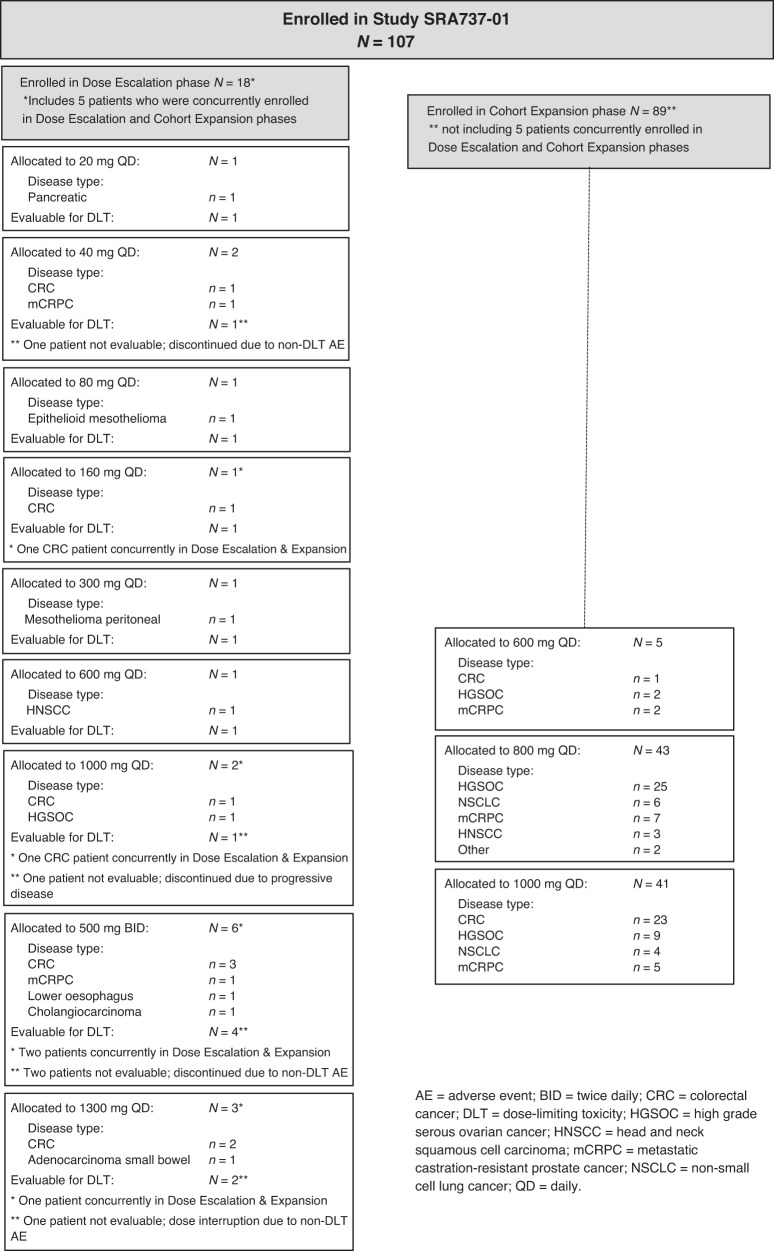
SRA737 monotherapy study enrolment by dose level. Patient numbers and tumour types enrolled in different cohorts in dose escalation and expansion phases of the study.

A total of 107 patients were enrolled in the study, including 18 patients across 13 dose-escalation cohorts and 89 patients in expansion cohorts; though eligible for enrolment, no patients with anal cancer were enrolled in the study. Five patients were concurrently enrolled in both dose-escalation and expansion cohorts (Fig. 1).

In the Dose Escalation phase the patients described as having other disease included pancreatic cancer (1), mesothelioma (2), esophageal carcinoma (1) and cholangiocarcinoma (1). In the Expansion Phase the patients described as having other disease included fallopian tube carcinoma (1) and oropharyngeal carcinoma (1).

The median age of patients in this study was 64 years (range 38–86 years) and the most common tumour types were HGSOC (*n* = 37), CRC (*n* = 32), and mCRPC (*n* = 16). Within the study, 98% (105/107), 41% (44/107) and 36% (38/107) had previously received chemotherapy, immunotherapy or radiotherapy respectively. In addition,100% (16/16) and 24% (9/37) of patients with prostate cancer and ovarian cancer had previously received hormonal therapy and PARP inhibitors respectively. More than 50% of patients had received four or more lines of previous treatment (Supplementary Table 2).

Patients enrolled in tumour-type expansion cohorts were required to have genetic alterations hypothesized to confer sensitivity to Chk1 inhibition. Of the pre-specified alterations required for eligibility, the most frequently detected were tumour suppressors TP53 (56% of patients), PTEN (15% of patients) and CDKN2A/B (8% of patients); DNA damage response genes BRCA2 (8% of patients) and ATM (7% of patients); and oncogenic drivers KRAS (23% of patients), FBXW7 (9% of patients), and MYC (8% of patients).

### Safety profile

#### Dose escalation and tolerability

The study enrolled a total of 18 patients in the dose-escalation phase and followed a single-patient dose escalation strategy at SRA737 dose levels of 20, 40, 80, 160, 300, 600 and 1000 mg QD. No DLTs were seen in the 1000 mg QD dose-escalation cohort (or any lower-dose cohorts), and thus the expansion cohorts were opened. Safety data were reviewed when a total of 16 patients had been enrolled at the 1000 mg QD dose level (dose-escalation or expansion cohort); of the 10 evaluable patients, 3 patients required dose reduction to 600 mg due to GI intolerability and 7 patients tolerated the 1000 mg dose but required the support of anti-emetic medication and/or night-time dosing. A cohort evaluating the 1000 mg daily dose split into two doses (500 mg twice daily [BID]) then enrolled six patients, four of whom were evaluable for DLT. One patient in the 500 mg BID dose group had a DLT of Grade 4 thrombocytopenia, and review of all patients showed no clear differentiation in overall tolerability of 500 mg BID vs. 1000 mg QD dosing, thus there was no further enrolment in BID dosing. Three patients were enrolled into the 1300 mg QD cohort; 2 of these patients had dose-limiting GI intolerability (Grade 1–2 nausea, vomiting, and/or diarrhoea), and 1 patient had dose-limiting Grade 3 neutropenia. Therefore, the dose of 1000 mg QD was considered the MTD. Expansion cohorts were initially enrolled at 600 mg QD (5 patients), however, the expansion phase was predominantly enrolled at the 1000 mg QD and 800 mg QD dose levels (Fig. 1).

### Overall safety profile

The most common treatment-emergent adverse events (TEAEs) attributed to SRA737 irrespective of SRA737 dose level were diarrhoea (63%), nausea (60%), vomiting (46%), fatigue (38%), decreased appetite (18%), neutropenia (16%) and anaemia (15%) (Table 1). Among the 49 patients treated at SRA737 doses >800 mg QD, the most frequent Grade 3 or higher TEAEs (aside from disease progression) related to SRA737 were neutropenia (10%), rash/rash maculopapular (6% combined), and fatigue (4%) (Table 2).

**Table 1:** SRA737-related treatment-emergent adverse events of any grade reported by ≥10% of overall patient group.

Preferred term	SRA737 dose ≤ 800 mg (*N* = 58) *n* (%)	SRA737 dose > 800 mg (*N* = 49) *n* (%)	Overall (*N* = 107) *n* (%)
Any SRA737-related treatment-emergent adverse event (TEAE)	51 (87.9)	47 (95.9)	98 (91.6)
Diarrhoea	33 (56.9)	34 (69.4)	67 (62.6)
Nausea	32 (55.2)	32 (65.3)	64 (59.8)
Vomiting	21 (36.2)	28 (57.1)	49 (45.8)
Fatigue	14 (24.1)	27 (55.1)	41 (38.3)
Decreased Appetite	12 (20.7)	7 (14.3)	19 (17.8)
Neutropenia	10 (17.2)	7 (14.3)	17 (15.9)
Anaemia	5 (8.6)	11 (22.4)	16 (15.0)

**Table 2:** SRA737-related treatment-emergent adverse events of Grade 3 or higher reported by 2 or more patients overall.

Preferred term	SRA737 dose ≤ 800 mg (*N* = 58) *n* (%)	SRA737 dose > 800 mg (*N* = 49) *n* (%)	Overall (*N* = 107) *n* (%)
Any SRA737-related Grade ≥3 treatment-emergent adverse event (TEAE)	14 (24.1)	19 (38.8)	33 (30.8)
Neutropenia	4 (6.9)	5 (10.2)	9 (8.4)
Lymphocyte count decreased	3 (5.2)	0	3 (2.8)
Rash	2 (3.4)	1 (2.0)	3 (2.8)
Rash maculopapular	1 (1.7)	2 (4.1)	3 (2.8)
Thrombocytopenia	2 (3.4)	1 (2.0)	3 (2.8)
Aspartate aminotransferase increased	2 (3.4)	0	2 (1.9)
Diarrhoea	1 (1.7)	1 (2.0)	2 (1.9)
Dyspnoea	2 (3.4)	0	2 (1.9)
Fatigue	0	2 (4.1)	2 (1.9)

Cardiac safety parameters were monitored closely in this study since cardiac side effects have been seen previously with Chk1 inhibitors. The studied doses had no clinically relevant effects on heart rate, PR interval, or QRS duration and there were no individual subjects with treatment-emergent QTcF values >500 ms or a change from baseline in QTcF (ΔQTcF) of >60 ms on central ECG monitoring. Four cardiac serious adverse events were evaluated to be possibly related to SRA737. At 800 mg SRA737, 1 case of asymptomatic myocardial infarction was diagnosed by the detection of elevated troponins on routine monitoring concurrent with ECG changes and mild systolic dysfunction and apical akinesis on echocardiogram, and 1 case of acute coronary syndrome with complaints of chest pain associated with elevated troponin but no ischemic changes noted on ECG occurred. At 1000 mg SRA737, 1 case of cardiomyopathy manifested by chest pain and shortness of breath with echocardiographic findings of left ventricular systolic dysfunction and asymptomatic myocardial infarction, and 1 case of asymptomatic QT prolongation with no cardiac signs or symptoms reported including during a period of in-hospital observation occurred. In all cases, administration of the drug was permanently discontinued. Based on an analysis of the relationship of SRA737 plasma concentration and QT interval corrected for heart rate using Fridericia’s formula (QTcF) measured in 79 patients, the QTcF least squares (LS) mean change from baseline (ΔQTcF) was similar across all dose groups and indicated that SRA737 has an effect on QTcF. The largest LS mean ΔQTcF at the recommended Phase 2 dose level of 800 mg QD was observed at 2 h post dose on Cycle 1 Day 22, corresponding to the time of maximum plasma concentration (*T*_max_) of SRA737, and reached 16.8 ms (90% confidence interval: 11.53 to 22.13). As noted above, no clinically significant treatment-emergent QTcF values and no clinically significant changes from Baseline were noted in a review of QTcF data from centrally read ECGs. Furthermore, no cases where ECG data demonstrated an increase in QTcF were associated with an arrhythmia or were associated with other symptoms. These clinical data reveal no indication that SRA737 is proarrhythmic at therapeutic doses.

A review of echocardiogram data obtained on 83 evaluable patients revealed that no additional patients (other than the cases mentioned above of clinically evident cardiomyopathy and myocardial infarction) had a > 10% absolute drop from baseline resulting in abnormal left ventricular ejection fraction.

Adverse events (AE) leading to treatment discontinuation were reported by a total of 34 patients (31.8%), 16 of whom (15.0%) discontinued due to SRA737-related TEAEs. Fatal adverse events AE were reported for ten patients; none were attributed to SRA737.

### Pharmacokinetic profile

The PK profile of SRA737 after repeated doses of 600, 800 and 1000 mg QD is presented in Table 3. The *T*_max_ of SRA737 generally occurred at between 2 and 4 h but ranged between 1 and 6 h. Estimated elimination half-life (*t*_½_) values ranged from 6.23 to 10.4 h, individual apparent total clearance of the drug from plasma after oral administration (CL/F) values ranged from 18.4 L/h to 119 L/h, and individual apparent volume of distribution (Vd/F) values ranged from 215 to 1590 L. Systemic exposure, as estimated by maximum plasma concentration (*C*_max_) and area under the plasma concentration-time curve (AUC), tended to be comparable between 600 mg and 1000 mg dose levels.

**Table 3:** Pharmacokinetic parameters (mean ± standard deviation) for plasma SRA737 following repeated doses.

Day	Dose (mg)	*N*(patients)	*t*_max_^a^ (h)	*C*_max_ (ng/mL)	*C*_min_ (ng/mL)	AUC_0-24_ (ng h/mL)	*t*_½_ (h)	CL/F (L/h)	Vd/F (L)
Repeat doses on C1D8, C1D22, C2D8, C2D15 or C2D22	600	7	2 (2–2)	2470 ± 328	230 ± 123	22100 ± 2450	8.22 ± 1.27	26.9 ± 2.85	320 ± 61.1
	800	31	2 (2–4)	2480 ± 679	312 ± 166	24700 ± 8580	8.25 ± 1.17	38.0 ± 22.2	454 ± 299
	1000	16	2 (1–6)	2490 ± 666	499 ± 185	27200 ± 9210	8.45 ± 1.06	39.7 ± 9.20	477 ± 87.0

*AUC*_*0-24*_  area under the curve 0 to 24 h, *C1*   Cycle 1, *CL/F*   apparent total clearance of the drug from plasma after oral administration, C_*max*_   maximum plasma concentration, C_*min*_   minimum plasma concentration, *D8*   Day 8 (of cycle), *h* hour, *ID*   insufficient data, *NA*   not available, *PK*   pharmacokinetics, *t*½ elimination half-life, *t*_max_   time of maximum plasma concentration, *Vd/F*   apparent volume of distribution.

^a^Median (minimum-maximum).

Upon repeated dosing at 800 mg and 1000 mg QD, systemic exposure generally remained similar regardless of specific sampling day (Cycle 1 Day 22 [C1D22], C1D8, C2D8, C2D15, or C2D22) compared to the first single dosing at Day −7 to Day −4, with individual accumulation ratio (R_AUC_) values ranging from 0.611 to 2.11.

**Fig. 2:**
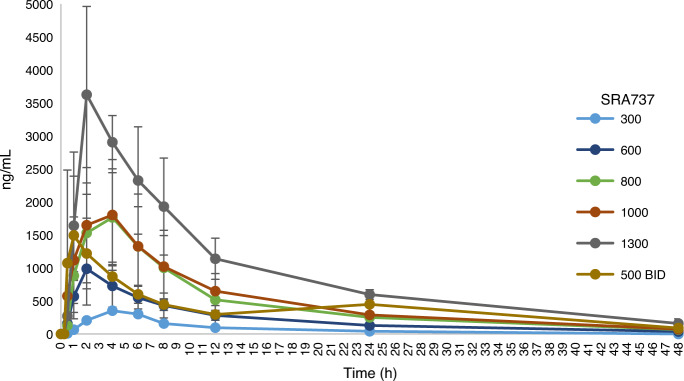
Single dose pharmacokinetic profile of SRA737. Mean plasma concentrations of SRA737 across different dose levels over a 48 hour sampling window. Note: Error bars indicate ±1 standard deviation.

The mean minimum SRA737 plasma concentration (*C*_min_) at the recommended Phase 2 dose of 800 mg QD was 312 ng/mL, which exceeded the minimally effective concentration of 100 nM (37.9 ng/mL) previously shown to inhibit Chk1 in preclinical experiments. All patients at or above the dose level of 300 mg QD achieved this concentration for at least 24 h following a single SRA737 dose, and up to 48 h for the majority of patients (Fig. 2).

### Determination of recommended Phase 2 dose

The majority of patients in this study initiated SRA737 at either the MTD of 1000 mg total daily dose (44/107 patients) or a dose of 800 mg QD (43/107 patients). The choice of 800 mg QD as the recommended Phase 2 dose for SRA737 monotherapy was based on the clinical observation that GI tolerability appeared to be improved at 800 mg QD versus the MTD of 1000 mg QD. Looking specifically at the frequency of AEs at 800 mg and 1000 mg SRA737, Grade 1–2 TEAEs of diarrhoea, nausea, and vomiting occurred in 68%, 78%, and 50% of patients at the 800 mg SRA737 dose level respectively, whilst these occurred in 72%, 65%, and 63% of patients respectively at the 1000 mg SRA737 dose level. Events of GI toxicity above Grade 2 were observed in one patient at the 800 mg dose level who experienced Grade 3 diarrhoea, whereas at the 1000 mg dose level 1 patient experienced Grade 3 diarrhoea and another single patient experienced both Grade 3 nausea and vomiting.

Exposure at both 800 mg QD and 1000 mg QD exceeded the minimum effective concentration extrapolated from preclinical models.

### Tumour response

**Fig. 3:**
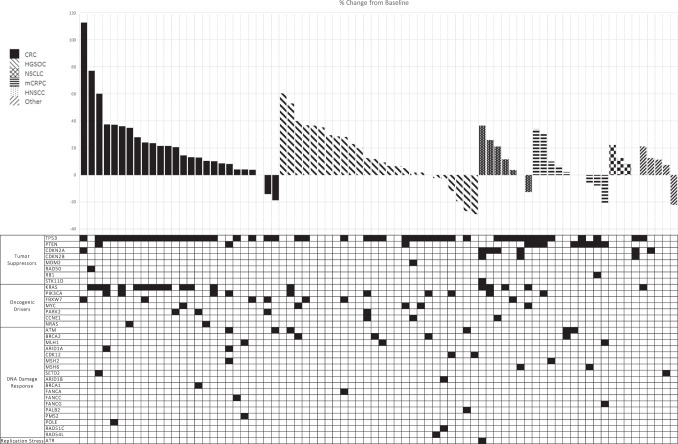
Clinical efficacy of SRA737. A waterfall plot of change in size of tumours represented as percentage at baseline in patients treated on the expansion cohorts. The genomic profile of individual patients are shown on the chart below the waterfall plot.

Efficacy analysis was performed on expansion cohort patients with predefined genetic mutations who received at least 75% of the planned dose of SRA737 in Cycle 1; there were no complete or partial RECIST responses (Fig. 3). The disease control rate, defined as patients who received at least four cycles of therapy with at least stable disease as their best response, was 8/25 (33.3%) in CRC, 11/26 (42.3%) in HGSOC, 3/8 (37.5%) in NSCLC, 8/13 (61.5%) in mCRPC, and 3/4 (75.0%) in HNSCC.

The lack of partial or complete RECIST responses precluded evaluation of associations between tumour response and genetic alterations, however, genetic aberrations of patients in expansion cohorts are displayed in Fig. 3.

## Discussion

Multiple Chk inhibitors have been evaluated in Phase 1/2 trials. The development of an early intravenously administered Chk1 inhibitor AZD7762 was halted due to concerns about cardiac toxicity including a drop in ejection fraction [9]. The oral Chk1 inhibitors GDC-0425 and GDC-0575 [10, 11] were evaluated in clinical trials which predominantly treated patients in combination cohorts with gemcitabine, with limited studies as a monotherapy available for GDC-0575. The Chk inhibitor LY2606368/prexasertib was administered intravenously and was further evaluated for efficacy as a single agent [12, 13].

Haematological toxicities have been limiting for most Chk1 inhibitors when evaluated as single agents or in combination with standard dose gemcitabine. Treatment-related haematological toxicity such as neutropenia of NCI CTCAE Grade 3 or higher occurred in 60% of patients with GDC-0575 in combination with standard dose gemcitabine [11] and ranged from 38% to 65% with prexasertib [14, 15], and Phase 2 studies of prexasertib have routinely used myeloid growth factor support [13]. It is therefore a significant advantage that Grade 3 or higher neutropenia occurred at a rate of only 8% in the current study. Interestingly, GI side effects were the most common adverse events related to SRA737 and the limiting factor for SRA737 dosing, with reports of diarrhoea (63% of patients), nausea (60%), and vomiting (46%); however, these events were reported at Grade 3 or higher severity by less than 5% of patients treated at the recommended Phase 2 dose of 800 mg QD or higher. It is also worth noting that the GI adverse effects were able to be controlled to a significant degree with either prophylactic or on treatment use of anti-emetics and anti-diarrheal agents. The reported rates for treatment-related GI adverse events of all grades in the study of the oral Chk1 inhibitor GDC-0575 in combination with standard dose gemcitabine included diarrhoea 18%, nausea 43%, and vomiting 27% [11]. The intravenous Chk1 inhibitor prexasertib was reported to show rates of diarrhoea, nausea, and vomiting at 20, 36 and 27%, respectively, in a Phase 2 study [14]. Since cardiac side effects were previously reported for AZD7762, ECG and echocardiographic assessments were carried out in this study of SRA737. Two patients had an ischemic cardiac event (1 myocardial infarction and 1 acute coronary syndrome) while on study, and thus a relationship to study drug could not be excluded; however, there were no commonly reported symptoms such as angina that would suggest myocardial ischemia across a wider cohort of patients. One patient had a reported drop of left ventricular ejection fraction from 65 to 20–25% on an echocardiogram performed outside the study. This type of effect has been reported with AZD7762 [9] but was not reported in patients receiving GDC-0575 or prexasertib used as a single agent. Thus, the toxicity profile of SRA737 as a single agent has differences and similarities with other Chk1 inhibitors; though neutropenia was seen at the non-tolerated dose of 1300 mg SRA737, and treatment-related neutropenia occurred in 16% of overall patients receiving SRA737, it was less frequently observed than with other oral or intravenous Chk1 inhibitors. Gastrointestinal toxicity, however, was reported at equal or higher rates for daily dosing with SRA737 compared with other CHK1 inhibitors such as GDC-0575 and intermittently-dosed prexasertib.

This study was designed to enrich expansion cohorts with mutations that could theoretically sensitize cells to Chk1 inhibitors but did not result in identifying subpopulations of patients whose disease responded sufficiently to SRA737. Some reductions in tumour size (up to 29% reduction) were seen, however, there were no partial or complete RECIST responses observed in the study and therefore rigorous evaluation of associations between tumour response and genetic alterations was not possible. Interestingly, in the group of 29 patients with evidence of antitumour effect via a reduction or no increase in sum of RECIST target tumour diameters and/or stable disease for 4 or more cycles, a number of potential associations with genetic alterations were noted. For example, alterations in the oncogenic driver genes FBXW7 (a negative regulator of cyclin E, encoded by CCNE1) and CCNE1 were observed in 6 patients across multiple tumour types (CRC, HGSOC, NSCLC). PIK3CA, another frequently modified oncogenic driver gene, was observed in four patients. Similarly, alterations in DNA damage response genes such as ATM, CDK12, and those of the RAD family (RAD51C, RAD54L) were observed across multiple tumour types. Of note, a previously tested Chk inhibitor, GDC-0575, did not report partial or complete responses when used as a single agent [11]. Prexasertib, an intravenously administered Chk1/2 inhibitor, did demonstrate efficacy in the form of partial responses in squamous anal and head and neck cancers in a Phase 1 study [12] and a 33% response rate in patients with high-grade serous ovarian cancers [13]. The dose levels of prexasertib used to achieve these response rates required support with granulocyte colony stimulating factor (G-CSF) for bone marrow toxicity. Pharmacokinetic modelling in this study suggested that SRA737 doses of 300 mg QD produced plasma concentrations consistent with concentrations in preclinical experiments showing Chk1 inhibition, and at this dose level, SRA737 was well tolerated. The findings related to the majority of populations/biomarkers studied in this trial did not show sufficient single agent activity to support further development of SRA737 as monotherapy. Given its mechanism of action resulting in abrogation of DNA damage repair, further clinical development of SRA737 should utilise combination therapy approaches. This study has successfully characterized the safety and PK profile of SRA737 given as monotherapy, which will be helpful in designing future combination studies. Indeed, a combination study of SRA737 with low-dose gemcitabine including expansion cohorts of patients with tumours harbouring selected genetic alterations hypothesized to confer sensitivity to Chk1 inhibition has been completed (NCT02797977).

In the combination study [16] the recommended Phase 2 dose was determined to be 500 mg SRA737 when combined with low-dose (250 mg/m2) gemcitabine. SRA737 in combination with low-dose gemcitabine was well tolerated with lower myelotoxicity than has been seen at standard doses of gemcitabine or with other combinations of Chk1 inhibitors with gemcitabine. RECIST partial tumour responses were observed in anogenital cancer, cervical cancer, high-grade serous ovarian cancer, rectal cancer, and small cell lung cancer.

## Supplementary information

Supplemental Material to SRA737 Manuscript

## Data Availability

Anonymized datasets generated and/or analysed during the current study are available from the corresponding author on reasonable request.

## References

[1] Sanchez Y, Wong C, Thoma RS, Richman R, Wu Z, Piwnica-Worms H (1997). Conservation of the Chk1 checkpoint pathway in mammals: linkage of DNA damage to Cdk regulation through Cdc25. Science.

[2] Pilie PG, Tang C, Mills GB, Yap TA (2019). State-of-the-art strategies for targeting the DNA damage response in cancer. Nat Rev Clin Oncol.

[3] Liu Q, Guntuku S, Cui XS, Matsuoka S, Cortez D, Tamai K (2000). Chk1 is an essential kinase that is regulated by Atr and required for the G(2)/M DNA damage checkpoint. Genes Dev.

[4] Smith HL, Southgate H, Tweddle DA, Curtin NJ (2020). DNA damage checkpoint kinases in cancer. Expert Rev Mol Med.

[5] Osborne JD, Matthews TP, McHardy T, Proisy N, Cheung KM, Lainchbury M (2016). Multiparameter lead optimization to give an oral checkpoint kinase 1 (CHK1) inhibitor clinical candidate: (R)-5-((4-((morpholin-2-ylmethyl)amino)-5-(trifluoromethyl)pyridin-2-yl)amino)pyr azine-2-carbonitrile (CCT245737). J Med Chem.

[6] Walton MI, Eve PD, Hayes A, Henley AT, Valenti MR, De Haven Brandon AK (2016). The clinical development candidate CCT245737 is an orally active CHK1 inhibitor with preclinical activity in RAS mutant NSCLC and Emicro-MYC driven B-cell lymphoma. Oncotarget.

[7] Rogers RF, Walton MI, Cherry DL, Collins I, Clarke PA, Garrett MD (2020). CHK1 inhibition is synthetically lethal with loss of B-family DNA polymerase function in human lung and colorectal cancer cells. Cancer Res.

[8] Zangarini M, Berry P, Sludden J, Raynaud FI, Banerji U, Jones P (2017). Development and validation of a LC-MS/MS method for the quantification of the checkpoint kinase 1 inhibitor SRA737 in human plasma. Bioanalysis.

[9] Sausville E, Lorusso P, Carducci M, Carter J, Quinn MF, Malburg L (2014). Phase I dose-escalation study of AZD7762, a checkpoint kinase inhibitor, in combination with gemcitabine in US patients with advanced solid tumors. Cancer Chemother Pharmacol.

[10] Infante JR, Hollebecque A, Postel-Vinay S, Bauer TM, Blackwood EM, Evangelista M (2017). Phase I study of GDC-0425, a checkpoint kinase 1 inhibitor, in combination with gemcitabine in patients with refractory solid tumors. Clin Cancer Res.

[11] Italiano A, Infante JR, Shapiro GI, Moore KN, LoRusso PM, Hamilton E (2018). Phase I study of the checkpoint kinase 1 inhibitor GDC-0575 in combination with gemcitabine in patients with refractory solid tumors. Ann Oncol.

[12] Hong D, Infante J, Janku F, Jones S, Nguyen LM, Burris H (2016). Phase I study of LY2606368, a checkpoint kinase 1 inhibitor, in patients with advanced cancer. J Clin Oncol.

[13] Lee JM, Nair J, Zimmer A, Lipkowitz S, Annunziata CM, Merino MJ (2018). Prexasertib, a cell cycle checkpoint kinase 1 and 2 inhibitor, in BRCA wild-type recurrent high-grade serous ovarian cancer: a first-in-class proof-of-concept phase 2 study. Lancet Oncol.

[14] Konstantinopoulos PA, Lee J-M, Gao B, Miller R, Lee J-Y, Colombo N, et al. A phase 2 study of prexasertib (LY2606368) in platinum resistant or refractory recurrent ovarian cancer. Gynecol Oncol. 2022;167:213–25. 10.1016/j.ygyno.2022.09.019 PMC10673677 36192237

[15] Byers LA, Navarro A, Schaefer E, Johnson M, Özgüroğlu M, Han J-Y (2021). A phase II trial of prexasertib (LY2606368) in patients with extensive-stage small-cell lung cancer. Clin Lung Cancer.

[16] Jones R, Plummer R, Moreno V, Carter L, Roda D, Garralda E (2023). A phase I/II trial of oral SRA737 (a Chk1 Inhibitor) given in combination with low-dose gemcitabine in patients with advanced cancer. Clin Cancer Res.

[17] Plummer ER, Kristeleit RS, Cojocaru E, Haris N, Carter L, Jones H (2019). A first in human phase I/II trial of SRA737 (a Chk1 Inhibitor) in subjects with advanced cancer. Am Soc Clin Oncol.

