# Supplementary material for: A Phase 1/2 trial of SRA737 (a Chk1 inhibitor) administered orally in patients with advanced cancer
Source: Br J Cancer. 2023 Apr 29;129(1):38–45. doi: 10.1038/s41416-023-02279-x (PMC10307885; doi:10.1038/s41416-023-02279-x)
Supplement: Supplementary file 1 — Supplemental Material to SRA737 Manuscript [file 41416_2023_2279_MOESM1_ESM.docx]

SUPPLEMENTARY MATERIAL

Supplementary Table 1: Genetic predictors of SRA737 sensitivity

A combination of gene mutations documented or predicted to enhance sensitivity to Chk1 inhibition/loss is required for enrolment into the cohort expansion phase of this study. These genes of interest are grouped into four main classes, consistent with the Hallmarks of Cancer (Hanahan, D., Weinberg, R. A. Hallmarks of cancer: the next generation. Cell144, 646–674).

| **Tumor Suppressor** |  | **DNA Damage Repair** | | | |  | **Replicative Stress** |  | **Oncogenic Driver** |
| --- | --- | --- | --- | --- | --- | --- | --- | --- | --- |
|  |  |  | | | |  |  |  |  |
| CDKN1A |  | ARID1A | FANCE | PALB2 | RAD54L |  | ATR |  | CCNE1^2^ |
| CDKN1B |  | ATM | FANCF | PMS2 | RPA1 |  | CHEK1 |  | FBXW7^3^ |
| CDKN2A |  | BLM | FANCG | POLD1 | SETD2 |  | Other |  | HRAS |
| CDKN2B |  | BRCA1 | FANCI | POLE | SMARCA4 |  |  |  | KRAS |
| CDKN2C |  | BRCA2 | FANCL | RAD50 | TP53BP1 |  |  |  | NRAS |
| RB1 |  | CDK12 | FANCM | RAD51 | XRCC2 |  |  |  | MYC |
| STK11 |  | CHEK2 | MLH1 | RAD51B | XRCC3 |  |  |  | MYCN |
| TP53 |  | FANCA | MRE11A | RAD51C | Other |  |  |  | PARK2 |
| MDM2^1^ |  | FANCC | MSH2 | RAD51D |  |  |  |  | PIK3CA |
| Other |  | FANCD2 | MSH6 | RAD52 |  |  |  |  | Other |
|  |  |  |  |  |  |  |  |  |  |
|  |  |  |  |  |  |  |  |  |  |
|  |  |  |  |  |  |  |  |  |  |
| 1. Amplification or gain of function mutations are desired for this gene 2. *CCNE1* gene amplification (or alternative genetic alteration with similar functional effect) is required for the *CCNE1* gene amplification-specific HGSOC cohort 3. Loss of function mutations are desired for this gene | | | | | | | | | |

Other genetic predictors could be added to this list, including mutations meeting any of the following criteria:

- A new gene/mutation that has been identified and published in at least 1 peer reviewed article documenting its relationship or sensitivity to genetic alterations with a Chk1 or ATR mutation
- Data from patient-derived xenograft (PDX) studies performed by the Sponsor or its collaborator demonstrating evidence of genetic sensitivity.
- Data of similar quality that has been reviewed by the Sponsor but is not yet published or conducted by the Sponsor or their collaborator.
- Detection of microsatellite instability in a tumor sample may increase the probability of detecting a germline mutation in a DNA mismatch repair gene. Five mononucleotide repeat markers (BAT-25, BAT-26, NR-21, NR-24, and MONO-27) are used to determine microsatellite instability.

Source: SRA737-01 Study Protocol Appendix 6

Supplementary Table 2: Demography of study SRA737-01

|  | **CRC (N = 32)** | **HGSOC (N = 37)** | **NSCLC (N = 10)** | **mCRPC (N = 16)** | **HNSCC (N = 4)** | **Other (N = 8)** | **Overall (N = 107)** |
| --- | --- | --- | --- | --- | --- | --- | --- |
|  | n (%) | n (%) | n (%) | n (%) | n (%) | n (%) | n (%) |
| **Age (years)** | | | | | | | |
| Mean (StD) | 61.3 (12.05) | 60.6 (9.81) | 64.4 (9.11) | 68.3 (6.76) | 63.0 (6.06) | 58.4 (6.46) | 62.2 (10.04) |
| Median (Q1, Q3) | 64.0 (52.0, 71.0) | 60.0 (55.0, 68.0) | 66.0 (59.0, 71.0) | 68.0 (65.0, 73.5) | 63.5 (58.0, 68.0) | 57.0 (53.0, 63.0) | 64.0 (56.0, 69.0) |
| Min, Max | 38, 79 | 39, 86 | 50, 75 | 54, 80 | 56, 69 | 52, 69 | 38, 86 |
| **Lines of Therapy^a^, n (%)** | | | | | | | |
| 1 | 0 | 0 | 0 | 0 | 1 (25.0) | 0 | 1 (0.9) |
| 2 | 4 (12.5) | 4 (10.8) | 4 (40.0) | 0 | 1 (25.0) | 1 (12.5) | 14 (13.1) |
| 3 | 10 (31.3) | 8 (21.6) | 2 (20.0) | 3 (18.8) | 2 (50.0) | 4 (50.0) | 29 (27.1) |
| 4 | 12 (37.5) | 9 (24.3) | 3 (30.0) | 0 | 0 | 2 (25.0) | 26 (24.3) |
| 5 | 4 (12.5) | 4 (10.8) | 0 | 5 (31.3) | 0 | 0 | 13 (12.1) |
| 6+ | 1 (3.1) | 12 (32.4) | 1 (10.0) | 7 (43.8) | 0 | 0 | 21 (19.6) |
| Not Reported | 1 (3.1) | 0 | 0 | 1 (6.3) | 0 | 1 (12.5) | 3 (2.8) |
| CRC = colorectal cancer, HGSOC = high grade serous ovarian cancer, HNSCC = head and neck squamous cell carcinoma, max = maximum, mCRPC = metastatic castration‑resistant prostate cancer, min = minimum, NSCLC = non‑small cell lung cancer, Q1 = first quartile, Q3 = third quartile, QD = daily, StD = standard deviation  ^a^ Based on the last anticancer therapy before enrollment | | | | | | | |

Supplementary methods

Study inclusion criteria

**Dose Escalation Phase and Cohort Expansion Phase**

1. Written (signed and dated) informed consent and capable of co-operating with treatment and follow up.

2a. For subjects in the Dose Escalation Phase: any locally advanced or metastatic, histologically or cytologically proven solid tumor or non-Hodgkin lymphoma that is relapsed after or progressing despite conventional treatment for which no conventional therapy is considered appropriate by the investigator or is declined by the subject.

2b. For subjects in the Cohort Expansion Phase: locally advanced or metastatic, histologically or cytologically proven malignancy of the types specified in inclusion criterion 10, for which no other conventional therapy is considered appropriate by the investigator or has been declined by the subject.

1. Life expectancy of at least 12 weeks.
2. World Health Organization (WHO) performance status of 0–1.
3. Hematological and biochemical indices within the ranges shown below, measured within one week prior to the subject receiving their first dose of investigational medicinal product.

| **Laboratory Test** | **Lower acceptable limit** |
| --- | --- |
| Hemoglobin | ≥ 90 g/L |
| Absolute neutrophil count | ≥ 1.5 x 10^9^/L |
| Platelet count | ≥ 100 x 10^9^/L |
| Bilirubin | ≤ 1.5 x upper limit of normal (ULN) unless due to Gilbert’s syndrome in which case up to 3 × ULN is permissible |
| Alanine aminotransferase and/or aspartate aminotransferase and Alkaline Phosphatase | ≤ 2.5 x ULN unless raised due to tumor in which case up to 5 x ULN is permissible |
| Serum Creatinine | ≤ 1.5 x ULN |

1. Attained the age of 18 years at the time consent is given.
2. Subjects must have archival tumor tissue available for tumor profiling or accessible tumor and willingness to consent to a biopsy for the collection of tumor tissue.

**Cohort Expansion Phase**

1. Subjects must have measurable disease (per Response Evaluation Criteria in Solid Tumors, version 1.1 [RECIST v1.1]) or, for metastatic castration-resistant prostate cancer (mCRPC), evaluable disease per any of the following:
2. Measurable disease per RECIST v1.1;
3. Increasing prostate specific antigen ; or
4. Circulating tumor cell count of 5 or more cells per 7.5 mL of blood.
5. Subjects must have tumor tissue or circulating tumor DNA evidence that their tumor harbors a combination of mutations which are expected to confer sensitivity to Chk1 inhibition. Eligibility will be determined by the Sponsor's review of genetic abnormalities detected in genes in the following categories:
6. Key tumor suppressor genes regulating G1 cell cycle progression/arrest such as RB1, TP53, etc. For patients with head and neck squamous cell carcinoma (NHSCC) or squamous cell carcinoma of the anus (SCCA), positive human papillomavirus (HPV) status is also considered for eligibility.
7. The DNA damage response pathway including ATM, BRCA1, and BRCA2. For patients with colorectal cancer (CRC), mismatch repair deficiency (MMR) genetic alterations and/or high microsatellite instability are also considered for eligibility.
8. Genetic indicators of replicative stress such as gain of function/amplification of Chk1 or ATR or other related gene.
9. Oncogenic drivers such as MYC, KRAS, etc.
10. Subjects must meet one of the following criteria (a-e):
    1. Metastatic CRC, defined by the following:

- Histologically and/or cytologically confirmed CRC, and
- Must have received at least 1 prior regimen for advanced/metastatic disease
  1. High-grade serous ovarian cancer (HGSOC), defined by the following:
- Histologically confirmed high-grade serous ovarian, fallopian tube or primary peritoneal cancer, and
- Recurrent platinum-intolerant subjects, or those with platinum-resistant disease, defined as radiological evidence of disease progression within 6 months of the last receipt of platinum-based chemotherapy. Patients with platinum refractory disease are not eligible.
  1. Advanced non-small cell lung cancer (NSCLC), defined by the following:
- Locally advanced and recurrent or metastatic, histologically confirmed NSCLC, and
- Must have received at least 1 prior regimen for advanced/metastatic disease
  1. mCRPC, defined by the following:
- Histologically or cytologically confirmed adenocarcinoma of the prostate that has progressed after androgen deprivation therapy
  1. HNSCC or SCCA, defined by the following:
- Histologically confirmed HNSCC from any primary site, or SCCA:
  - For HNSCC: locally advanced disease (ie, persistent or progressive disease following curative-intent radiation, and not a candidate for surgical salvage due to incurability or morbidity), or metastatic disease
  - For SCCA: locally advanced disease or metastatic disease for which no curative intent therapy is available
- Must have received at least 1 prior regimen for advanced/metastatic disease

**Study exclusion criteria**

1. Have received the following prior or current anticancer therapy:
   1. Radiotherapy within the last 6 weeks (except for symptom control and where the lesions will not be used as measurable disease)
   2. Endocrine therapy during the previous 4 weeks except for luteinizing hormone releasing hormone agonists for prostate cancer
   3. Chemotherapy during the previous 4 weeks
   4. Immunotherapy during the previous 6 weeks
   5. Nitrosoureas or Mitomycin C during the previous 6 weeks
   6. Other IMPs during the 4 weeks before treatment
   7. Any prior treatment with a Chk1 inhibitor, or prior treatment with an ATR inhibitor within 6 months prior to receiving SRA737.
2. Other malignancy within the past 2 years with the exception of adequately treated tumors that are associated with an expected 5‑year disease-free survival of approximately 95% or better.
3. Ongoing toxic manifestations of previous treatments greater than National Cancer Institute Common Terminology Criteria for Adverse Events [NCICTCAE] Grade 1. Exceptions to this are alopecia or certain toxicities, which in the opinion of the investigator and the sponsor or sponsor’s designee should not exclude the subject.

4a. For subjects in the Dose Escalation Phase that are not to be included in an Expansion Cohort: new or progressing brain metastases. Subjects with brain metastases that have been radiologically stable over an 8-week period may be included in this phase.

4b. For subjects in the Cohort Expansion Phase: present or prior brain metastases.

1. Women of childbearing potential or women who are already pregnant or lactating. However, those patients who have a negative serum or urine pregnancy test before enrollment and agree to use two forms of contraception as defined in the protocol or agree to sexual abstinence, effective from the first administration of SRA737, throughout the trial and for six months afterwards are considered eligible.
2. Male subjects with partners of childbearing potential (unless they agree to take measures not to father children by using a barrier method of contraception as defined in the protocol from the first administration of SRA737 through the trial and for 6 months after their final SRA737 dose). Men with pregnant or lactating partners must be advised to use barrier method contraception (eg, condom plus spermicidal gel) to prevent exposure of the fetus or neonate.
3. Major surgery from which the subject has not yet recovered.
4. At high medical risk because of non-malignant systemic disease including active uncontrolled infection.
5. Known to be serologically positive for hepatitis B, hepatitis C or human immunodeficiency virus.
6. Serious cardiac condition, such as concurrent congestive heart failure, prior history of class III/ IV cardiac disease (New York Heart Association), left ventricular ejection fraction < 45% at baseline, history of cardiac ischemia within the past 6 months, or prior history of cardiac arrhythmia requiring treatment.
7. Prior bone marrow transplant or extensive radiotherapy to greater than 25% of bone marrow within 8 weeks.
8. Peanut allergy.
9. QT interval corrected for heart rate using Fridericia’s formula (QTcF) > 450 msec in adult males and > 470 msec in adult females.
10. Impairment of gastrointestinal (GI) function or GI disease that may significantly alter the absorption of SRA737 (eg, ulcerative diseases, uncontrolled nausea, vomiting, diarrhea, or malabsorption syndrome).
11. Not able to swallow capsules without chewing or crushing.
12. Is a participant or plans to participate in another interventional clinical trial, whilst taking part in this Phase 1/2 study of SRA737. Participation in an observational trial or interventional clinical trial which does not involve administration of an investigational medicinal product and which would not place an unacceptable burden on the subject in the opinion of the investigator and Sponsor or Sponsor’s designee would be acceptable.
13. Any other condition which in the investigator’s opinion would not make the subject a good candidate for the clinical trial.

Dose Limiting Toxicity Criteria

The DLT window is defined from the first dose (Day -7 to Day -4) until the end of Cycle 1 (up to 35 days).

A DLT is defined as any highly probably or probably SRA737-related event of:

- Grade 4 neutropenia or thrombocytopenia that lasts for > 7 days despite withholding dosing and/or providing supportive care (eg, hematopoietic growth factors)

Note: In the event of a Grade 4 neutropenia or Grade 4 thrombocytopenia, a full blood count must be performed at least on Day 7 after the onset of the event to determine if a DLT has occurred. The investigator must continue to monitor the subject closely until resolution to ≤ Grade 3.

- Febrile Neutropenia
- A ≥ Grade 3 thrombocytopenia with ≥ Grade 3 bleeding
- A ≥ Grade 3 nonhematological toxicity with the following possible exceptions per Cohort Review discussion:
  - Alopecia of any grade
  - Grade 3 or 4 nausea or vomiting in subjects that have not received optimal treatment with anti-emetics
  - Grade 3 or 4 diarrhea in subjects that have not received optimal treatment with anti-diarrheal medication
  - Transient, asymptomatic Grade 3 biochemical abnormalities if agreed by the Medical Monitor and the Chief Investigator
  - Grade 3 fatigue, unless there is an increase by at least 2 grades from baseline (classed as the grade prior to first dose)
- Inability to receive 75% of planned dose in the first cycle during the DLT window due to drug-related toxicity
